# Genomic Analysis of the Unusual Staphylococcus aureus ST630 Isolates Harboring WTA Glycosyltransferase Genes *tarM* and *tagN*

**DOI:** 10.1128/spectrum.01501-21

**Published:** 2022-02-16

**Authors:** Mengyuan Xiong, Liangjun Chen, Jin Zhao, Xiao Xiao, Junying Zhou, Fang Fang, Xinwei Li, Yunbao Pan, Yirong Li

**Affiliations:** a Department of Laboratory Medicine, Zhongnan Hospital of Wuhan University, Wuhan, China; b Medical School of Zhengzhou University, Zhengzhou, China; c Hubei Engineering Center for Infectious Disease Prevention, Control and Treatment, Wuhan, China; University of Calgary

**Keywords:** *Staphylococcus aureus*, whole genome sequencing, wall teichoic acid, glycosyltransferase, horizontal gene transfer, mobile genetic elements

## Abstract

Staphylococcus aureus (S. aureus) can cause a broad spectrum of diseases ranging from skin infections to life-threatening diseases in both community and hospital settings. The surface-exposed wall teichoic acid (WTA) has a strong impact on host interaction, pathogenicity, horizontal gene transfer, and biofilm formation in S. aureus. The unusual S. aureus ST630 strains containing both ribitol-phosphate (RboP) WTA glycosyltransferase gene *tarM* and glycerol-phosphate (GroP) WTA glycosyltransferase gene *tagN* have been found recently. Native PAGE analysis showed that the WTA of *tagN, tarM*-encoding ST630 strains migrated slower than that of *non-tagN-encoding* ST630 strains, indicating the differences in WTA structure. Some mobile genetic elements (MGEs) such as the unique GroP-WTA biosynthetic gene cluster (SaGroWI), SCC*mec* element, and prophages that probably originated from the CoNS were identified in *tagN, tarM*-encoding ST630 strains. The SaGroWI element was first defined in S. aureus ST395 strain, which was refractory to exchange MGEs with typical RboP-WTA expressing S. aureus but could undergo horizontal gene transfer events with other species and genera via the specific bacteriophage Φ187. Overall, our data indicated that this rare ST630 was prone to acquire DNA from CoNS and might serve as a novel hub for the exchange of MGEs between CoNS and S. aureus.

**IMPORTANCE** The structure of wall-anchored glycopolymers wall teichoic acid (WTA) produced by most Gram-positive bacteria is highly variable. While most dominant Staphylococcus aureus lineages produce poly-ribitol-phosphate (RboP) WTA, the *tagN, tarM*-encoding ST630 lineage probably has a poly-glycerol-phosphate (GroP) WTA backbone like coagulase-negative staphylococci (CoNS). There is growing evidence that staphylococcal horizontal gene transfer depends largely on transducing helper phages via WTA as the receptor. The structural difference of WTA greatly affects the transfer of mobile genetic elements among various bacteria. With the growing advances in sequencing and analysis technologies, genetic analysis has revolutionized research activities in the field of the important pathogen S. aureus. Here, we analyzed the molecular characteristics of ST630 and found an evolutionary link between ST630 and CoNS. Elucidating the genetic information of ST630 lineage will contribute to understanding the emergence and diversification of new pathogenic strains in S. aureus.

## INTRODUCTION

Staphylococcus aureus (S. aureus), a notorious Gram-positive bacterial pathogen, can cause a broad spectrum of diseases ranging from superficial skin, soft tissue infections to life-threatening pneumonia, sepsis, and endocarditis (1–4). The great threat of S. aureus infections to human beings is mainly because of the rapid emergence of antibiotic resistant and highly virulent isolates (5). The major S. aureus clones that cause infectious diseases worldwide are reported to belong to several pandemic lineages. The horizontal gene transfer (HGT) of mobile genetic elements (MGEs) enabled S. aureus to constantly evolve and is the main means to transfer genetic information (DNA) among and within bacterial species (6). Like other bacteria, S. aureus possesses various MGEs such as transposons, phages, plasmids, pathogenicity islands, and the staphylococcal cassette chromosome *mec* (SCC*mec*) element, which confers β-Lactam family of antibiotics resistance in methicillin-resistant S. aureus (MRSA) (7). Clonal complex 1 (CC1) and CC8 have been reported to contain various community-associated MRSA (CA-MRSA) strains, which commonly carry relatively small SCC*mec* elements, which confer methicillin resistance and the phage-encoded toxin Panton-Valentine leukocidin (PVL) (8, 9). ST239 is recognized as a common lineage in bloodstream infections and is part of CC8 but has gained a large DNA fragment from CC30. CC5 is found worldwide and is sometimes pandemic. In addition, ST630 has recently been reported to cause severe infective endocarditis in China (10). CC398 has been increasingly reported as a cause of invasive infections in patients (11). The distribution of MGEs is highly variable, suggesting that “short-distance” HGT of MGEs occurs among closely related bacteria at a high frequency due to the specific recognition of cognate recipient strains. However, the “long-distance” HGT across species boundaries also occurs between different species or even genera at a lower frequency (12).

Genetic and biochemical analysis on several staphylococcal phages revealed that staphylococcal HGT of MGEs is primarily dependent on bacteriophage-mediated transduction, which uses the species- or strain-specific wall teichoic acid (WTA) as the major receptor (13, 14). The majority of S. aureus lineages produce a classical ribitol-phosphate (RboP) WTA, modified with d-alanine, α- and/or β-O-*N*-acetylglucosamine (GlcNAc) (15). The *tarM* gene is responsible for the glycosylation of WTA with α-GlcNAc at the O-4 position of the ribitol residue, whereas *tarP* and *tarS* are responsible for the β-GlcNAc glycosylation at O-3 and O-4 position, respectively (16–18). Interestingly, the *tarS* gene is conserved in almost all S. aureus strains, whereas *tarM* is absent in several certain CCs (CC5, CC398, CC45, etc.). Compared to S. aureus, coagulase-negative staphylococci (CoNS) strains usually produce glycerophosphate (GroP) WTA, modified with variable sugar substituents. It has been a mystery how phage-mediated HGT of genetic materials between S. aureus and other CoNS is accomplished. To note, an unusual S. aureus ST395 lineage has been reported to synthesize CoNS-like WTA, which is composed of GroP backbone modified with α-*N*-acetyl-d-galactosamine (α-GalNAc) residues. ST395 isolates contain a novel genetic element named S. aureus GroP WTA island (SaGroWI), consisting of several transposon-related sequences and four genes (*tagV*, *tagF*, *tagD*, and *tagN*), that are responsible for biosynthesis and α-GalNAc glycosylation of GroP WTA (19). ST395 strains are prohibited from undergoing phage-dependent HGT events, with typical RboP-WTA expressing S. aureus due to this special WTA structure, but they have access to MGEs from CoNS species via specific bacteriophage Φ187 (20).

We have previously reported a rare *S aureus* lineage ST630 harboring both *tarM* and *tagN* genes (21), suggesting that it may produce GroP-GalNAc WTA and RboP-GlcNAc WTA simultaneously. Previous studies generally focused on the molecular epidemiology of S. aureus ST630 clones, with little respect to the complete genomes and WTA biosynthesis genes (22, 23). Here, we determined the draft genome sequences of a group of S. aureus ST630 strains and included them in comparative genomic analyses with other Staphylococci, mainly focusing on various MGEs and WTA biosynthesis and modification genes.

## RESULTS

### Genome sequencing, assembly, and annotation.

**FIG 1:**
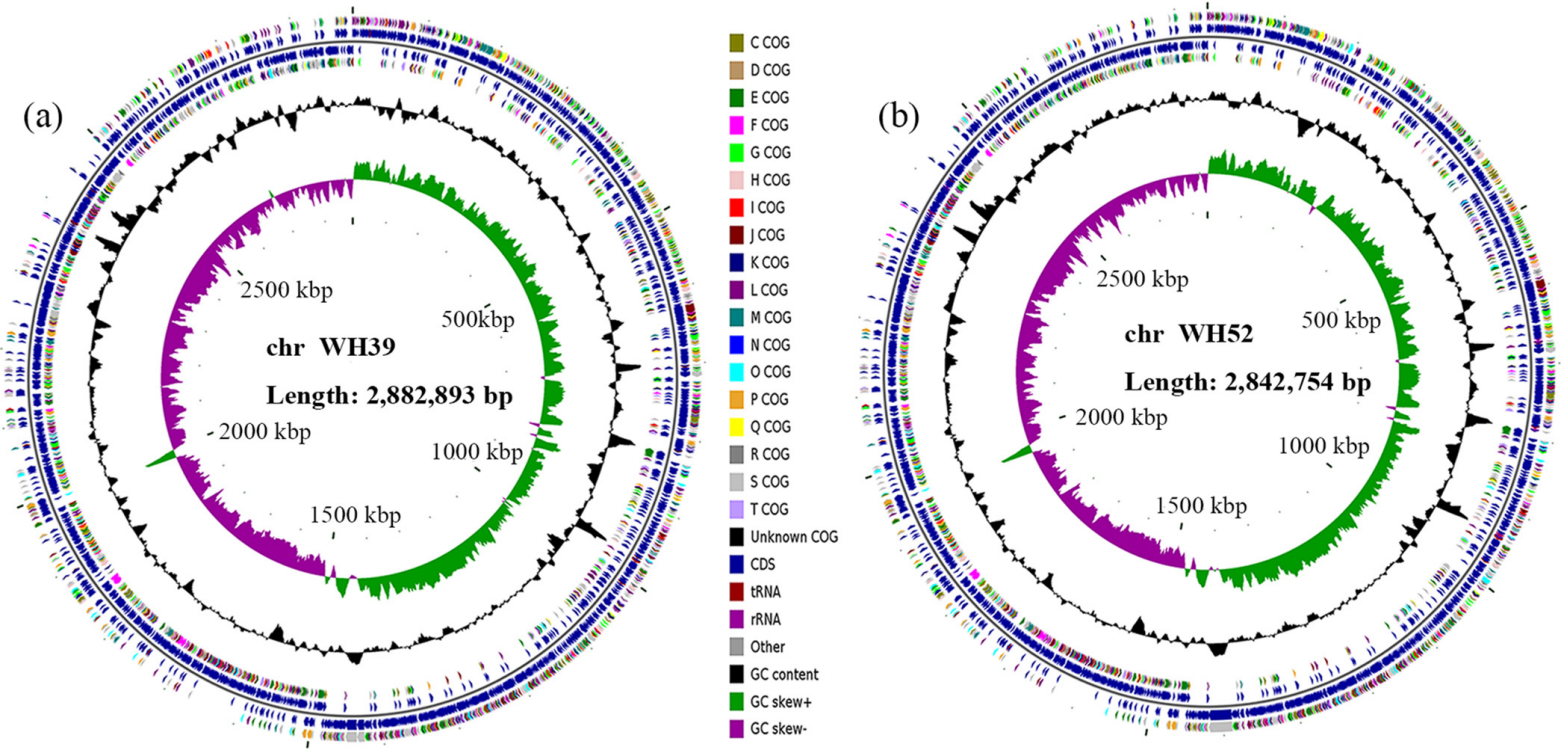
Circular maps of the strain WH39 (a) and WH52 (b) chromosomes. From the inner to outer circle: the first (innermost) circle represents the scale, the second circle represents GC skew, the third circle represents the GC content, the fourth and seventh circles represent the COG of each CDS, and the fifth and sixth circles represent the positions of CDS, tRNA, and rRNA on the genome.

General genome and assembly characteristics of 16 clinical S. aureus isolates collected in the present study are shown in Table 1. The genomes of WH39 (*tagN*-encoding) and WH52 (non-*tagN*-encoding) were closed, with all reads united into a single circular chromosome. The genome circular maps of WH39 and WH52 chromosomes are shown in Fig. 1. The unclosed genomes of 14 other S. aureus isolates were assembled into 28 to 530 contigs. The genome sizes of 16 isolates varied from 2659666 bp to 3040523 bp, with the GC contents ranging from 32.50% to 32.82%. The number of predicted coding sequences (CDS) ranged from 2,653 to 3,195. In addition, these genomes contained 3 to 16 rRNA, 19 to 60 tRNA, and 4 to 5 ncRNA genes.

**TABLE 1:** The genome and assembly characteristics of clinical S. aureus strains collected in the present study

Strain	Genome size (bp)	Contig no.	GC content	Genome coverage	Contig N50 (bp)	Longest contig (bp)	Shortest contig (bp)	CDSs	5S rRNAs	16S rRNAs	23S rRNAs	tRNAs	ncRNAs	Accession no.
BJ12	2742597	49	32.60%	444.0x	130672	518539	971	2653	2	1	1	42	4	JACSCT000000000
BJ95	2752796	58	32.55%	212.0x	130512	269920	535	2684	2	1	1	56	4	JAJNRL000000000
HN288	2814465	70	32.62%	443.0x	91859	251546	679	2775	2	1	1	42	4	JACSCV000000000
NXNE	3040523	361	32.50%	410.0x	31309	110219	769	3195	2	1	1	29	5	JACSCU000000000
NX98	2764714	47	32.61%	112.0x	128785	370569	542	2723	2	1	1	57	4	JAJNRM000000000
WH60	2756939	53	32.58%	434.0x	137934	459316	1143	2689	2	1	1	42	4	JACSCK000000000
WH99	2793342	43	32.61%	388.0x	141243	459061	769	2748	2	1	1	42	4	JACSCL000000000
WH114	2852635	60	32.70%	402.0x	129031	339786	934	2798	2	1	1	42	4	JACSCM000000000
WH119	2819585	56	32.59%	415.0x	120247	370596	769	2796	2	1	1	34	4	JACSCN000000000
WH211	2717727	421	32.67%	413.0x	10262	39903	239	2935	1	1	1	19	4	JACSCO000000000
WH299	2771230	56	32.63%	409.0x	128999	331897	290	2719	2	1	1	42	4	JACSCP000000000
WH17	2730179	28	32.82%	427.0x	178484	465206	1071	2668	3	1	2	43	4	JACSCQ000000000
WH31	2864942	60	32.68%	441.0x	89206	310920	1141	2829	2	1	1	34	4	JACSCR000000000
WH231	2659666	530	32.71%	416.0x	7660	31367	242	2907	2	1	2	37	4	JACSCS000000000
WH39	2882893	1	32.70%	432.0x				2828	6	5	5	60	4	CP060491
WH52	2842754	1	32.74%	331.0x				2760	6	5	5	58	4	CP060584

### Phylogenetic analysis and pulsed-field gel electrophoresis (PFGE).

**FIG 2:**
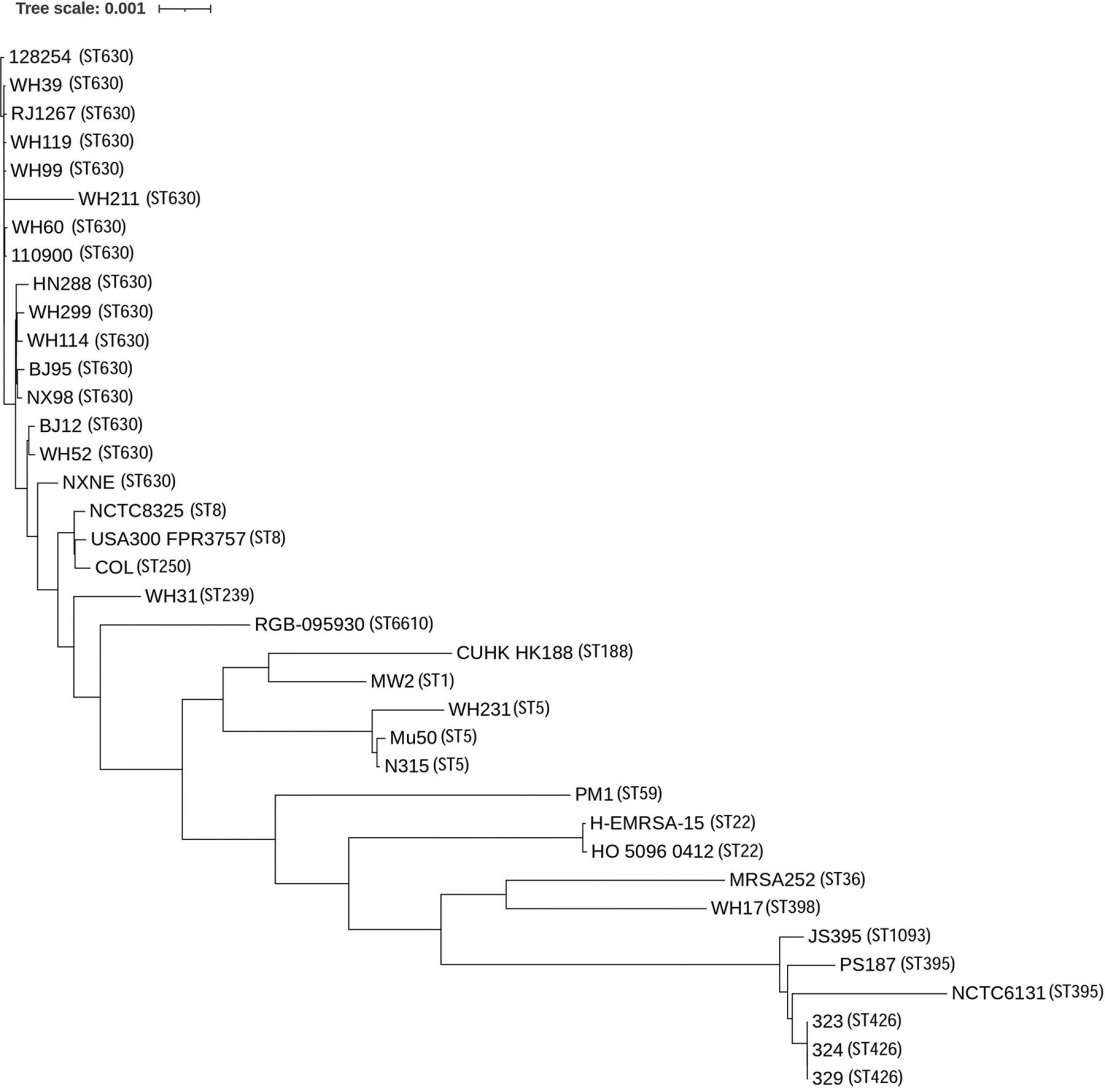
Phylogenetic tree based on 1711 single copy orthologous genes extracted from 37 S. aureus genomes.

A total of 37 genome sequences of S. aureus isolates were included in the phylogenetic analysis. By proteins prediction and homology search, 1,711 putative orthologous genes were identified in all the 37 isolates and presented a single copy in each isolate. The phylogenetic tree inferred from 1,711 putative orthologous genes suggested that ST395, ST1093, and ST426 branched deeply from other ST lineages (Fig. 2). Moreover, the genetic relatedness between ST630 and other S. aureus lineages was closer than that of ST395. A dendrogram of PFGE patterns among the 13 clinical ST630 isolates was shown in Fig. S1. Eleven PFGE types were detected and were named PFGE 01 to PFGE 11, with the similarity coefficients ranging from 82.7% to 100.0%. PFGE 03 contained 3 strains, and the other PFGE types contained 1 strain each. As derived from the dendrogram, different ST630 isolates exhibited over 80% similarity in DNA fragment patterns, with some variation in the size of certain DNA bands, indicating that there was a close genetic relationship among them.

### Most ST630 isolates have unique WTA biosynthesis genes and WTA structures.

**FIG 3:**
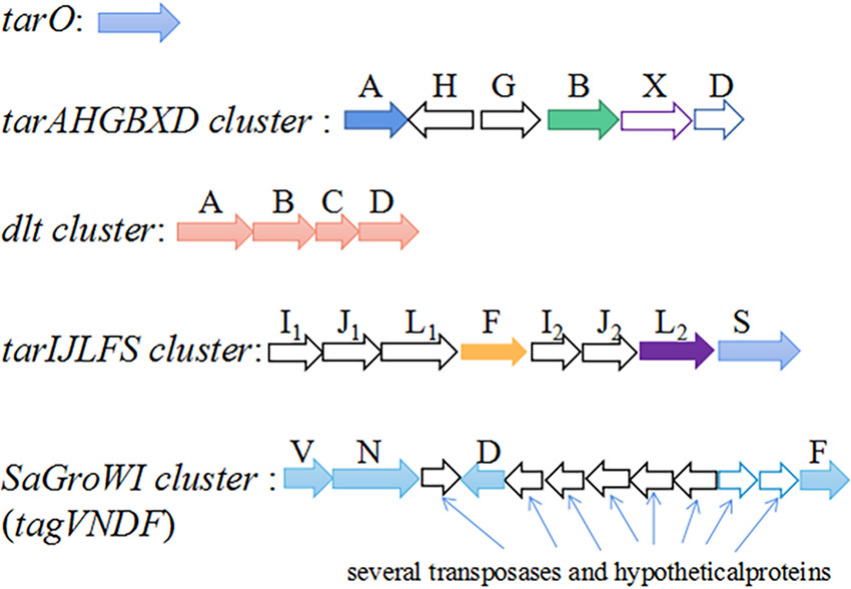
WTA biosynthesis genes found in the genomes of ST630.

Similar to most RboP-WTA expressing S. aureus strains, all the 16 ST630 isolates contained the *tarO* gene and the *tarAHGBXD* gene cluster for WTA linkage unit biosynthesis and WTA transport. The RboP-α-GlcNAc glycosyltransferase gene *tarM* was present in all 16 ST630 strains. BJ12 and WH52 also harbored the *tarIJLFS* cluster for RboP WTA polymerization and β-GlcNAc glycosylation. Notably, WH39, WH60, WH99, WH114, WH119, WH211, WH299, HN288, NX98, BJ95, RJ1267, 110900, and 128254 contained a SaGroWI gene cluster, replacing the *tarIJLFS* cluster. The SaGroWI gene cluster has been identified to be responsible for the GroP WTA biosynthesis and α-GalNAc glycosylation (*tagN*) (19, 20). Surprisingly, the remaining isolate NXNE harbored both the SaGroWI gene cluster and *tarIJLFS* cluster (Fig. 3). The homologs of these genes were identified in multiple CoNS strains such as *S. carnosus* TM300, *S. lugdunensis* NCTC12217, S. hominis K1, *S. warnei* NCTC7291, S. epidermidis B1200343, S. pseudintermedius ED99, etc. (Fig. S2 in the supplemental material).

**FIG 4:**
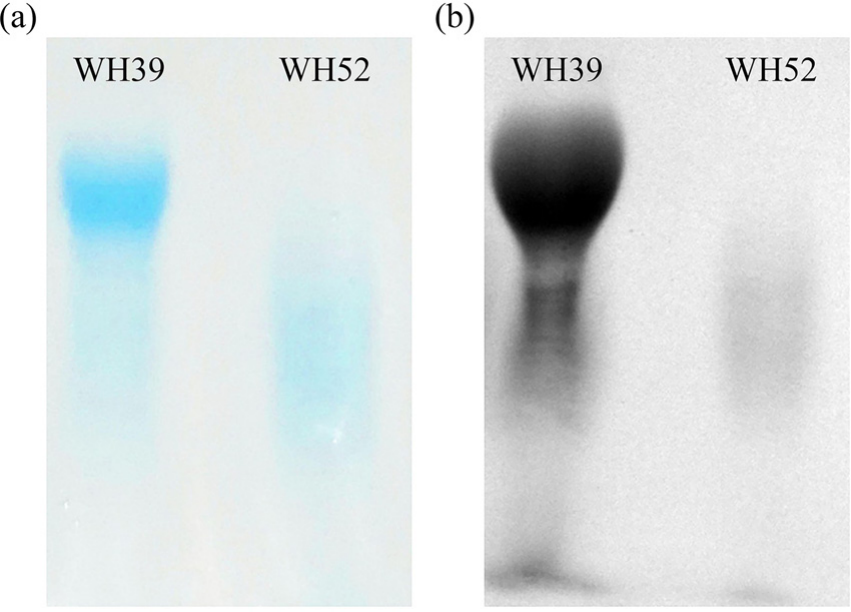
Native PAGE analysis of WTA preparations of S. aureus ST630 strains (WH39 and WH52) with two different glycosylation patterns. Samples were resolved in polyacrylamide gels and visualized with alcian blue (a)/silver (b) staining.

The crude WTA samples were applied to the native PAGE and visualized by Alcian blue-silver staining. The WTA of WH52 (non-*tagN*-encoding) migrated faster than that of WH39 (*tagN*-encoding), suggesting the structural difference in WTA (Fig. 4). Moreover, we constructed a *tagN* mutant (ΔTN) and its complementation (c-ΔTN) in WH39. The PAGE migration behaviors showed that the WTA of ΔTN migrated faster than that of the parental isolate, similar to that of the non-*tagN*-encoding ST630. The WTA of c-ΔTN migrated like that of the wild type, suggesting that ΔTN might indeed lack the WTA GalNAc residues (Fig. S3).

### A novel SCC*mec* element identified in WH39.

**FIG 5:**
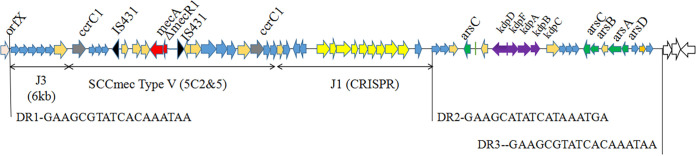
Genetic structure of the SCC*mec* element of strain WH39. The *ccrC* genes, *mecA* gene, and CRISPR genes are shown in gray, red, and yellow, respectively.

Distinct from the characterized SCC*mec* elements described previously, WH39 harbored a novel 59 kb SCC*mec* element that only had 55.42% homology with SCC*mec* type V (5C2&5) locating on CA-MRSA strain JCSC 5952 (GenBank accession number AB478780.1). This novel SCC*mec* element was inserted into the 3′ end of the *orfX* gene and flanked by two direct repeats (DR1 and DR3), containing 65 open reading frames (ORFs). Another DR2 was identified 38 kb downstream of the *orfX* gene, demarcating a two-domain composite element. The first 38 kb SCC region consisted of 40 ORFs, including a class-C2-like *mec* gene complex and two distinct *ccrC* genes (*ccrC1* allele 2 and *ccrC1* allele 8) (Fig. 5). Interestingly, the sequences of the *mec* gene complex and *ccr* gene exhibited high similarity to those found in the SCC*mec* elements of four CoNS strains, including Staphylococcus schleiferi strain TSCC54 (99.95%), Staphylococcus capitis strain CR01 (99.96%), Staphylococcus pseudintermedius strain AP20 (99.96%), and Staphylococcus haemolyticus strain PK-01 (99.94%), as well as S. aureus strain AR_0470 (99.97%). J1 region carried a clustered regularly interspersed short palindromic repeat (CRISPR) region, which was typically composed of repeat sequences interspersed with variable spacers and nine CRISPR-associated genes (cas1, cas2, cas10, csm2-6, and cas6). Fifteen spacers were identified in the CRISPR region and were most identical to CRISPR spacers in S. capitis CR01 (15/15 spacers), S. schleiferi TSCC54 (14/15 spacers), and S. aureus 08BA02176 (13/15 spacers). The last six spacers in WH39 exhibited 100% sequence identity to those identified in S. aureus JS395. BLAST searches showed that the sixth CRISPR spacer in WH39 was also identical to sequences from an S. aureus phage, phiIPLA-RODI, or Stab20.

The *mec*-associated direct repeat unit (*dru*) typing has been widely used to study the horizontal movement of the SCC*mec* element in S. aureus. The *dru*-type of WH39 was dt11ah (5a-2d-4a-0-2d-4f-3a-2g-3b-4e-3e), which differed slightly from *dru* types dt11c (5a-2d-4a-0-2d-5b-3a-2g-4b-4e-3e) found in *S. capitis* strain CR01, dt11ax (5a-2d-4a-0-2d-6f-3a-2g-3b-4e-3e) found in S. schleiferi strain TSCC54, dt11a (5a-2d-4a-0-2d-5b-3a-2g-3b-4e-3e) found in S. haemolyticus strain PK-01, and dt9v (5a-2d-4a-0-2d-2g-3b-4e-3e) found in S. aureus strain JS395.

### Prophages analysis.

Prophages prediction by PHASTER showed that all ST630 strains contained at least one prophage that ranged in length from 6.4 kb to 47.8 kb. The GC content varied between 26.06% and 35.28%. The features of these prophages are listed in Table S1. Based on the criteria of PHASTER, putative prophages with different completeness were identified in the present study. If the prophage region's total score was less than 70, it would be marked as incomplete; if between 70 and 90, it would be marked as questionable; if greater than 90, it would be marked as intact. Notably, a prophage closely related to the ST395-specific bacteriophage Φ187, which had the capacity to mediate HGT between S. aureus ST395 and other bacterial species, was only detected in all available genomes of *tagN*, *tarM-*encoding ST630 isolates, but not in *non-tagN*-encoding S. aureus ST630 strains WH52 and BJ12.Moreover, some common prophages, such as ΦPT1028 and ΦJay2Jay, were predicted in both *tagN*-encoding and non-*tagN*-encoding ST630 strains. Several WTA biosynthesis genes such as WTA translocase gene were observed in a 6.4 kb prophage of *tagN*, *tarM-*encoding ST630 isolates by BLAST analysis, providing further proof for the mobility of WTA biosynthetic genes.

## DISCUSSION

The S. aureus population belongs to many independent evolutionary lineages. As many virulence and resistance genes are encoded on MGEs, the HGT of MGEs promotes the genetic and phenotypic variation in S. aureus and has a strong impact on adaptation to changing environments. Recently, WGS and comparative analyses have been successfully applied to acquire the characteristics of bacteria and provide relevant information for understanding adaptation to host environment and mechanisms of pathogenicity (24, 25). There were occasional reports of ST630 in China, but most of them focused on molecular epidemiology, with few focusing on complete genome or WTA genes (10, 22). In the present study, we determined the genome sequences of previously collected S. aureus isolates and included them in the phylogenetic analysis with other *S. aureus* clones that represented a genetically diverse collection of strains of several different MLST types.

Of 16 ST630 isolates, 14 contained a SaGroWI gene cluster, the presence of which had previously been reported only in the ST395 strain. Additional genes involved in WTA biosynthesis initiation, translocation, d-alanylation, and α-GlcNAc glycosylation were present in ST630 strains. Winstel et al. (20) found that ectopic ST395 WTA genes expression in S. aureus RN4220 leads to the production of both RboP-GlcNAc WTA and GroP-GalNAc WTA, via NMR spectra analysis. Therefore, it is reasonable to speculate that *tagN*, *tarM*-encoding S. aureus ST630 strains can synthesize GroP-GalNAc WTA and RboP-GlcNAc WTA simultaneously. The PAGE migration behaviors showed WTA of WH39 had a slower migration than that of WH52, indicating the *tagN*-encoding ST630 had a more complex WTA structure. The origin of the SaGroWI elements previously found in ST395, and the exact timing and mechanism of their entry into ST630, remained unknown. Multiple transposase-related DNA fragments integrated into SaGroWI suggested that these WTA genes might be combined by rather recent recombination events and played a role in the evolution process. Notably, several CoNS share GroP-WTA biosynthesis genes (*tagVNDF*) homologs with ST630 strains, suggesting there is an evolutionary link between ST630 clones and CoNS. Some MGEs identified in *tagN*-encoding ST630 strains, were closely related to MGEs found in CoNS. The *mec*,*ccr* gene complexes and CRISPR/Cas region in the SCC*mec* element of WH39 exhibited high identity to those found in several CoNS and S. aureus strains, suggesting S. aureus ST630 can exchange DNA with CoNS and other S. aureus strains. Orthologs are usually genes encoding essential enzymes, coenzymes, or key regulatory proteins that are functionally conserved, and can change at a rate that covers the entire evolutionary history. The phylogenetic analysis based on single-copy homologous genes showed that the genetic relatedness between ST630 and other S. aureus lineages was closer than that of ST395. Taken together, the multiple shared DNAs and close relatedness indicate that the *tagN*, *tarM*-encoding ST630 can undergo HGT events with typical RboP-WTA expressing S. aureus and CoNS strains. Since WTA plays important roles in pathogen–host interactions and bacterial communication, the investigation of genome characteristics and WTA structures will enable assessment of the potential for HGT to cross species and genera boundaries in the future.

There were some limitations to the present study. First, the small sample size of ST630 strains limited the broad representativeness of this study. We will continue to expand the sample size in subsequent experiments. Second, WGS could only provide sequence information and predictions, but not actual WTA structures or gene transfer levels. Third, it remains unclear whether the unique WTA structure affects pathogenicity and immune responses, which will be the focus in further research.

In conclusion, the present study describes the unusual S. aureus ST630 isolates that may produce both RboP-GlcNAc WTA and GroP-GalNAc WTA. Comparative genome analyses suggest that this rare ST630 is prone to acquire DNA from CoNS and may serve as a gene transfer station between other S. aureus and CoNS species, thereby creating a possible new route for the interspecies HGT of various MGEs.

## MATERIALS AND METHODS

### Isolates.

Thirteen ST630, one ST398, one ST239, and one ST5 S. aureus isolates previously identified in our laboratory were sequenced in this study, which presented different WTA glycosyltransferase gene patterns as previously described (21). The molecular typing, WTA glycosyltransferase genes, and antibiotics susceptibility profiles are listed in Table S2. These non-duplicate isolates were collected from inpatients with infectious symptoms. All S. aureus isolates were identified by mass spectrometry analysis using matrix-assisted laser desorption/ionization time-of-flight (MALDI-TOF) mass spectrometry (Vitek MS, bioMérieux). All identified strains were stored at −80°C for further analysis.

### Antimicrobial susceptibility testing.

Antimicrobial susceptibility testing (AST) against oxacillin (OXA), penicillin (PEN), vancomycin (VAN), gentamicin (GEN), ciprofloxacin (CIP), levofloxacin (LEV), moxifloxacin (MXF), clindamycin (CLI), erythromycin (ERY), tetracycline (TET), quinupristin-dalfopristin (QUD), rifampicin (RIF), tigecycline (TGC), and linezolid (LZD) were conducted using Vitek2 compact (bioMérieux Inc., France). The control strains of AST were ATCC 25923, ATCC 29213, and ATCC 43300. The AST results were interpreted by the Clinical and Laboratory Standards Institute (CLSI) M100-S27.

### Genome sequencing, annotation, and comparative analysis.

Genomic DNA was extracted using a rapid DNA isolation kit (Aidlab, China). DNA libraries were prepared by next-generation sequencing using the Illumina NovaSeq platform (Illumina, San Diego, CA) with 2 × 150 bp paired-end reads. The *de novo* genome assemblies were generated with A5-MiSeq software (version 20160825) (26) and SPAdes software (version 3.12.0) (27). In addition, S. aureus WH39 and WH52 were also sequenced using the Pacbio RSII platform (Pacific Biosciences, Menlo Park, CA). The sequence data obtained by the Pacbio RSII platform were assembled using HGAP software (version 4) (28) and CANU software (version 1.7.1) (29). Base correction was performed using Pilon software (version 1.18) (30). Prediction and annotation of genes were performed using GeneMarkS software (version 4.32) (31) and BLAST software (version 2.5.0, https://blast.ncbi.nlm.nih.gov/Blast.cgi), respectively. The SCC*mec* typing, multilocus sequence typing (MLST), and *spa* typing identifications were performed using SCC*mec*Finder (version 1.2), MLST (version 2.0), and *spa*Typer (version 1.0) tools from the Center for Genomic Epidemiology (http://www.genomicepidemiology.org/). The rRNA and tRNA gene predictions were done using Barrnap software (version 0.9) and tRNAscan-SE software (version 1.3.1), respectively. The *mec*-associated direct repeat unit (*dru*) typing was assigned using a website (www.dru-typing.org), as previously described (32). The circular maps of chromosomes were created using the CGview server (http://cgview.ca) (33). The PHAge Search Tool Enhanced Release (PHASTER) web server (http://phaster.ca/) was used for the rapid prediction and annotation of prophage sequences (34). The putative CRISPR loci and Cas cluster were identified using the CRISPRCasFinder web server (https://crisprcas.i2bc.paris-saclay.fr/CrisprCasFinder/Index).

Twenty-one other available complete genome sequences of ST630, CC395, and other epidemic *S. aureus* clones published previously were obtained from the NCBI database for the phylogenetic analysis (Table S3) to represent a genetically diverse collection of strains of several different MLST types. (11, 35–38). Protein sequences of 37 S. aureus isolates were predicted using Prodigal software (version 2.6.3) for subsequent cluster analysis. The cluster analysis was performed by OrthoFinder software (version 2.3.8) to obtain single copy orthologous genes (39). These sequences were aligned using multiple sequence alignment program MAFFT (version 7), and FastTree (version 2.1) was used for the phylogenetic tree construction (40).

### Construction of *tagN* mutant and complementation.

The *tagN* mutant (ΔTN) in S. aureus WH39 was constructed by allelic replacement using the pKOR1 shuttle vector as described previously (41). The approximately 1-kb upstream and downstream fragments of the *tagN* gene were amplified separately by PCR using chromosomal DNA of S. aureus WH39 as a template. Primers used for gene deletion are listed in Table S4. The above amplification products were purified and spliced by fusion PCR, and then recombined on vector pKOR1 using the Gateway BP ClonaseTM II enzyme mix (Invitrogen Corp., CA, USA). Recombinant plasmid pKOR1-*ΔtagN* was transformed into S. aureus WH39 via electroporation to obtain the ΔTN strain. The allelic replacement mutant was checked by PCR and DNA sequencing. The S. aureus*-*E. coli shuttle vector pTSSCm was used to construct the expression of plasmid pTSSCm-*tagN* at BamHI and XhoI sites (primers; see Table S4) (42). The resulting constructs were then transformed into ΔTN via electroporation to obtain the *tagN* mutant complementation (c-ΔTN). The complementation was identified by PCR and DNA sequencing.

### Extraction and native PAGE analysis of WTA.

WTA was extracted with trichloroacetic acid (TCA) as described previously with some modifications (43). Briefly, S. aureus strains were grown in 200 mL tryptic soy broth (TSB) overnight at 35°C. Cells were collected by centrifugation at 4,500 rpm for 10 min and washed once with 30 mL of buffer 1 [50 mM 2-(N-morpholino)-ethanesulfonic acid (MES), pH = 6.5] and resuspended in 10 mL of buffer 2 [50 mM MES, 4% wt/vol sodium dodecyl sulfate (SDS), pH = 6.5]. After sonication for 20 min, the samples were incubated in a boiling water bath for 1 h. Then, the cells were collected by centrifugation at 12,000 rpm for 5 min, and the sediment was washed once with 2 mL of buffer 2, once with 2 mL of buffer 3 (50 mM MES, 2% wt/vol NaCl), and once with 2 mL of buffer 1. The sample was then resuspended in 2 mL of buffer 4 (0.5% wt/vol SDS, 20 mM Tris-HCl, 20 μg/mL proteinase K). After incubation at 70°C for 2 h, the pellets were washed with 3 mL of buffer 3 and then washed four times with ultrapure water to move SDS. The sample was finally resuspended in 1 mL of buffer 5 (5%TCA) and incubated at 65°C for 5 h with shaking to release WTA. Peptidoglycan was moved by centrifugation at 14,000 rpm for 20 min, and the supernatant was stored as crude WTA at −80°C. The WTA was analyzed by native PAGE and visualized using an alcian blue-silver staining method as described previously (16, 44, 45).

### PFGE.

Chromosomal DNA was digested with SmaI restriction enzyme (New England BioLabs) as described previously (46). The samples were loaded on pulsed-field certified agarose (1%) (Bio-Rad) using a CHEF mapper system (Bio-Rad, Munich, Germany). A Salmonella serotype Braenderup strain (H9812) was used as the molecular size standard. Running parameters were as follows: initial switch, 5 s; final switch, 40 s; time, 20 h; voltage, 6 V/cm; and temperature, 14°C. Gels were stained with 500 mL ethidium bromide (EB) for 25 min, destained in water for 40 min, and viewed using the Gel doc XR imaging system to obtain electrophoretic images. The similarity coefficients of DNA banding patterns were analyzed using BioNumerics software (version 4.0, Applied Maths, Ghent, Belgium). Similarity coefficients of 100% were determined as the same PFGE type, and those less than 100% were determined as different PFGE type. A similarity cutoff value of 80% was set to define the PFGE cluster. Isolates exhibiting identical DNA patterns were regarded as genotypically indistinguishable.

### Data availability.

The whole genome sequence data from this study are deposited in the NCBI database. The accession numbers can be found in Table 1. Additional information can be obtained from the corresponding author upon reasonable request.
