# Supplementary material for: Genomic Analysis of the Unusual Staphylococcus aureus ST630 Isolates Harboring WTA Glycosyltransferase Genes tarM and tagN
Source: Microbiol Spectr. 2022 Feb 16;10(1):e01501-21. doi: 10.1128/spectrum.01501-21 (PMC8849055; doi:10.1128/spectrum.01501-21)
Supplement: SUPPLEMENTAL FILE 1 — Supplemental material. Download SPECTRUM01501-21_Supp_1_seq6.pdf, PDF file, 1.5 MB [file spectrum01501-21_supp_1_seq6.pdf]

SUPPLEMENTAL MATERIAL

Table S1 List of prophages identified in ST630 *S. aureus* strains

Strain	Prophage region ID	Length	Completeness	Score	Total Proteins	Region Position	Most Common Phage	GC%
BJ12	Prophage 1	19.6Kb	incomplete	20	16	contig9:31298-50981	PHAGE_Staphy_StB20_like_NC_028821	33.33%
	Prophage 2	15.4Kb	questionable	75	21	contig20:33929-49348	PHAGE_Staphy_PT1028_NC_007045	31.74%
BJ95	Prophage 1	6.4Kb	incomplete	60	10	contig8:45120-51547	PHAGE_Strept_Jay2Jay_NC_029098	28.59%
	Prophage 2	22kb	incomplete	20	26	contig16:56-22019	PHAGE_Staphy_187_NC_007047	32.64%
HN288	Prophage 1	6.4Kb	questionable	70	9	contig12:13138-19571	PHAGE_Strept_Jay2Jay_NC_029098	28.57%
	Prophage 2	14.8Kb	incomplete	10	21	contig12:132814-147637	PHAGE_Staphy_PT1028_NC_007045	31.50%
	Prophage 3	35.6Kb	incomplete	40	58	contig41:1-35610	PHAGE_Staphy_187_NC_007047	34.64%
	Prophage 4	12.9Kb	incomplete	20	15	contig49:1620-14542	PHAGE_Staphy_PT1028_NC_007045	32.63%
NXNE	Prophage 1	42.9Kb	incomplete	50	64	contig38:64-43036	PHAGE_Staphy_phiN315_NC_004740	33.07%
	Prophage 2	27.5Kb	incomplete	60	28	contig47:272-27840	PHAGE_Staphy_phinm2_NC_028913	34.34%
	Prophage 3	22Kb	incomplete	10	26	contig52:141-22147	PHAGE_Staphy_187_NC_007047	34.94%
	Prophage 4	6.4Kb	questionable	70	9	contig86:663-7114	PHAGE_Strept_Jay2Jay_NC_029098	28.58%
	Prophage 5	11Kb	incomplete	20	31	contig104:1-11036	PHAGE_Staphy_96_NC_007057	34.26%
	Prophage 6	8.6Kb	incomplete	10	21	contig119:1-8652	PHAGE_Staphy_77_NC_005356	35.10%
NX98	Prophage 1	6.4Kb	incomplete	60	9	contig6:77239-83667	PHAGE_Strept_Jay2Jay_NC_029098	28.60%
	Prophage 2	30.5Kb	incomplete	40	25	contig8:70678-101202	PHAGE_Staphy_PT1028_NC_007045	32.87%
	Prophage 3	39.3Kb	questionable	90	65	contig22:66-39379	PHAGE_Staphy_187_NC_007047	34.33%
WH39	Prophage 1	15.4Kb	questionable	88	22	858247-873665	PHAGE_Staphy_PT1028_NC_007045	31.73%
	Prophage 2	7.9Kb	questionable	70	14	1577666-1585626	PHAGE_Bacter_Diva_NC_028788	27.30%
	Prophage 3	40.5Kb	incomplete	50	62	2262657-2303229	PHAGE_Staphy_187_NC_007047	34.47%
	Prophage 4	25.9Kb	intact	95	30	2518589-2544532	PHAGE_Staphy_PT1028_NC_007045	30.84%
	Prophage 5	6.4Kb	incomplete	50	7	2659030-2665474	PHAGE_Strept_Jay2Jay_NC_029098	28.58%

WH52	Prophage 1	46.4Kb	questionable	90	37	157565-204031	PHAGE_Staphy_SPbeta_like_NC_029119	30.29%
	Prophage 2	15.4Kb	questionable	75	21	824887-840306	PHAGE_Staphy_PT1028_NC_007045	31.72%
	Prophage 3	7Kb	incomplete	30	18	1544234-1551255	PHAGE_Strept_9871_NC_031069	26.06%
	Prophage 4	43.9Kb	intact	100	64	2228780-2272682	PHAGE_Staphy_53_NC_007049.1	34.26%
	Prophage 5	34.6Kb	questionable	88	28	2492095-2526707	PHAGE_Staphy_PT1028_NC_007045	32.12%
WH60	Prophage 1	6.4Kb	questionable	70	9	contig16:11529-17962	PHAGE_Strept_Jay2Jay_NC_029098	28.58%
	Prophage 2	15.4Kb	questionable	75	21	contig20:33936-49354	PHAGE_Staphy_PT1028_NC_007045	31.73%
	Prophage 3	19.6Kb	incomplete	20	16	contig5:60032-79678	PHAGE_Staphy_StB20_like_NC_028821	33.32%
	Prophage 4	22Kb	incomplete	35	25	contig4::21560-43678	PHAGE_Staphy_187_NC_007047	34.26%
WH99	Prophage 1	40.3Kb	intact	100	66	contig3:80112-120415	PHAGE_Staphy_187_NC_007047	34.42%
	Prophage 2	6.4Kb	incomplete	60	9	contig7:77316-83749	PHAGE_Strept_Annadreamy_NC_048719	28.58%
	Prophage 3	15.4Kb	questionable	75	22	contig20:4371-19789	PHAGE_Staphy_PT1028_NC_007045	31.71%
	Prophage 4	19.6Kb	incomplete	20	16	contig5:59976-79659	PHAGE_Staphy_StB20_like_NC_028821	33.33%
WH114	Prophage 1	40.7Kb	questionable	90	65	contig1:88751-129503	PHAGE_Staphy_187_NC_007047	34.49%
	Prophage 2	13.5Kb	incomplete	20	16	contig1:325996-339512	PHAGE_Staphy_PT1028_NC_007045	33.22%
	Prophage 3	6.4Kb	incomplete	60	9	contig6:45196-51629	PHAGE_Prochl_P_SSM2_NC_006883	28.60%
	Prophage 4	13.6Kb	incomplete	20	16	contig29:1620-15226	PHAGE_Staphy_PT1028_NC_007045	32.40%
WH119	Prophage 1	40.5Kb	intact	100	67	contig4:80110-120695	PHAGE_Staphy_187_NC_007047	34.56%
	Prophage 2	6.4Kb	incomplete	60	9	contig6:77376-83809	PHAGE_Synech_ACG_2014f_NC_047714	28.58%
	Prophage 3	13.5Kb	incomplete	20	16	contig11:140-13656	PHAGE_Staphy_PT1028_NC_007045	33.23%
	Prophage 4	27.5Kb	incomplete	20	21	contig15:54354-81904	PHAGE_Staphy_PT1028_NC_007045	32.95%
WH211	Prophage 1	29.6Kb	incomplete	60	51	contig37:558-30191	PHAGE_Staphy_187_NC_007047	35.28%
	Prophage 2	15Kb	incomplete	20	22	contig55:4022-19099	PHAGE_Staphy_PT1028_NC_007045	31.39%
WH299	Prophage 1	39.7Kb	incomplete	50	64	contig1:85296-125021	PHAGE_Staphy_187_NC_007047	34.42%
	Prophage 2	6.4Kb	questionable	70	9	contig7:77374-83807	PHAGE_Strept_Jay2Jay_NC_02909	28.58%
	Prophage 3	19Kb	incomplete	20	11	contig12:58623-77625	PHAGE_Staphy_PT1028_NC_007045	33.44%

	Prophage 4	4.8Kb	incomplete	20	8	contig35:1022-5877	PHAGE_Staphy_PT1028_NC_007045	32.43%
RJ1267	Prophage 1	15.4Kb	questionable	88	22	812549-827967	PHAGE_Staphy_PT1028_NC_007045	31.73%
	Prophage 2	28.5Kb	intact	110	28	964920-993502	PHAGE_Escher_RCS47_NC_042128	29.68%
	Prophage 3	8.8Kb	questionable	70	15	1527910-1536768	PHAGE_Bacter_Sitara_NC_028854	27.17%
	Prophage 4	47.8Kb	intact	100	72	1973119-2020948	PHAGE_Staphy_P282_NC_048634	33.30%
	Prophage 5	22Kb	incomplete	20	24	2053456-2075462	PHAGE_Staphy_PT1028_NC_007045	31.61%
	Prophage 6	40.3Kb	intact	100	65	2274051-2314415	PHAGE_Staphy_187_NC_007047	34.45%
	Prophage 7	16.6Kb	intact	95	23	2540250-2556923	PHAGE_Staphy_PT1028_NC_007045	30.95%
	Prophage 8	6.4Kb	incomplete	40	7	2676385-2682829	PHAGE_Escher_500465_1_NC_049342	28.58%
128254	Prophage 1	15.4Kb	questionable	88	23	828388-843806	PHAGE_Staphy_PT1028_NC_007045	31.73%
	Prophage 2	7.9Kb	questionable	70	14	1317600-1325560	PHAGE_Paenib_Xenia_NC_028837	27.30%
	Prophage 3	29.7Kb	questionable	80	17	1866518-1896262	PHAGE_Escher_RCS47_NC_042128	29.91%
	Prophage 4	6.6Kb	incomplete	40	7	2019540-2026154	PHAGE_Staphy_phi7401PVL_NC_020199	30.99%
	Prophage 5	30.5Kb	incomplete	30	24	2022220-2052722	PHAGE_Staphy_PT1028_NC_007045	30.93%
	Prophage 6	40.5Kb	intact	100	65	2251193-2291728	PHAGE_Staphy_187_NC_007047	34.36%
	Prophage 7	16.6Kb	questionable	88	22	2519405-2536078	PHAGE_Staphy_PT1028_NC_007045	30.95%
	Prophage 8	6.4Kb	incomplete	50	8	2655501-2661927	PHAGE_Strept_Jay2Jay_NC_029098	28.58%
110900	Prophage 1	15.4Kb	questionable	88	22	858072-873490	PHAGE_Staphy_PT1028_NC_007045	31.73%
	Prophage 2	28.5Kb	intact	100	28	1010440-1039022	PHAGE_Escher_RCS47_NC_042128	29.67%
	Prophage 3	7.9Kb	questionable	70	14	1573275-1581235	PHAGE_Staphy_187_NC_007047	27.30%
	Prophage 4	47.8Kb	incomplete	60	68	2016647-2064514	PHAGE_Staphy_P282_NC_048634	33.22%
	Prophage 5	22Kb	incomplete	20	24	2097022-2119029	PHAGE_Staphy_PT1028_NC_007045	31.61%
	Prophage 6	16.6Kb	intact	95	23	2544405-2561078	PHAGE_Staphy_PT1028_NC_007045	30.95%
	Prophage 7	6.4Kb	incomplete	40	7	2680540-2686984	PHAGE_Strept_Comrade_NC_048728	28.58%

Region Length: The length of the sequence of that region (in bp).

Completeness: A prediction of whether the region contains a intact or incomplete prophage based on the above criteria.
Score: The score of the region based on the above criteria.
Intact: score > 90
Questionable: score 70-90
Incomplete: score < 70
Region Position: The start and end positions of the region on the bacterial chromosome.
Most Common Phage: The phage(s) with the highest number of proteins most similar to those in the region.
GC %: The percentage of GC nucleotides of the region.

Table S2 Molecular typing, WTA glycosyltransferase genes and antibiotics susceptibility of clinical *S.aureus* strains

**Strain**	**Source**	**CC**	***spa***	**ST**	***tarP***	***tarS***	***tarM***	***tagN***	**MRSA/ MSSA**	**LEV**	**CIP**	**MXF**	**GEN**	**PEN**	**OXA**	**VAN**	**ERY**	**CLI**	**LZD**	**TET**	**RIF**	**QUD**	**TGC**
BJ12	pharynx swab	8	t377	630	-	+	+	-	MSSA	R	R	I	S	R	S	S	R	R	S	S	S	S	S
BJ95	blood	8	t377	630	-	-	+	+	MSSA	S	S	S	S	R	S	S	S	S	S	S	S	S	S
HN288	pus	8	t377	630	-	-	+	+	MSSA	R	R	R	S	R	S	S	R	S	S	S	S	S	S
NXNE	tissue	8	t4549	630	-	+	+	+	MRSA	S	S	S	S	R	R	S	R	R	S	S	S	S	S
NX98	tissue	8	t4549	630	-	-	+	+	MSSA	S	S	S	S	R	S	S	R	R	S	S	S	S	S
WH39	wound secretion	8	t2196	630	-	-	+	+	MRSA	S	S	S	S	R	R	S	R	R	S	S	S	S	S
WH52	wound secretion	8	t377	630	-	+	+	-	MSSA	S	S	S	S	R	S	S	S	S	S	R	S	S	S
WH60	wound secretion	8	t4549	630	-	-	+	+	MRSA	S	S	S	S	R	R	S	R	R	S	S	S	S	S
WH99	wound secretion	8	t4549	630	-	-	+	+	MRSA	S	S	S	S	R	R	S	S	S	S	S	S	S	S
WH114	catheter tip	8	t4047	630	-	-	+	+	MSSA	S	S	S	S	R	S	S	R	R	S	R	S	S	S
WH119	blood	8	t4549	630	-	-	+	+	MRSA	S	S	S	S	R	R	S	S	S	S	S	S	S	S
WH211	wound secretion	8	t4549	630	-	-	+	+	MRSA	S	S	S	S	R	R	S	R	R	S	S	S	S	S
WH299	wound secretion	8	t377	630	-	-	+	+	MSSA	S	S	S	S	R	S	S	S	S	S	S	S	S	S

WH17	wound secretion	398	t034	398	-	+	-	-	MSSA	S	S	S	S	R	S	S	R	R	S	S	S	S	S
WH31	bronchoalveolar lavage fluid	8	t030	239	-	+	+	-	MRSA	R	R	R	R	R	R	S	S	S	S	R	R	S	S
WH231	wound secretion	5	t002	5	+	+	-	-	MSSA	S	S	S	S	R	S	S	S	S	S	S	S	S	S

R-resistant, I-intermediate, S-susceptible

BJ, Beijing Tsinghua Changgung Hospital

HN, Hainan General Hospital

NX, General Hospital Of Ningxia Medical University

WH, Zhongnan Hospital Of Wuhan University

Table S3 List of the ST630, CC395, and other epidemic *S. aureus* clones used in this study

**Strain**	**Sequence type**	**Clonal complex**	**Country**	**Accession number**
JS395	ST1093	CC395	Switzerland	NZ_CP012756
RJ1267	ST630	CC8	China	NZ_CP047321
PS187	ST395	CC395	Germany	ARPA00000000
PM1	ST59	CC59	Japan	NZ_BAFA00000000
Mu50	ST5	CC5	Japan	NC_002758
N315	ST5	CC5	N315	BA000018
NCTC8325	ST8	CC8	England	LS483365
MW2	ST1	CC1	Japan	NC_003923
MRSA252	ST1	CC1	England	NC_002952
COL	ST250	CC8	America	NC_002951
FPR3757	ST8	CC8	America	NC_007793
110900	ST630	CC8	Denmark	CP058615
128254	ST630	CC8	Denmark	CP058613

H-EMRSA-15	ST22	CC22	Belgium	NZ_CP007659
HO 5096 0412	ST22	CC22	England	HE681097
CUHK_HK188	ST188	CC1	China	JFFV00000000
RGB-095930	ST6610	CC8	Germany	CP077098
323	ST426	CC395	Germany	CP077906
324	ST426	CC395	Germany	CP077899
329	ST426	CC395	Germany	CP077894
NCTC6131	ST395	CC395	England	LR134268

Table S4 Primers used in the present study

**Primer**	**sequence (5' –3')**	**Application**
tagN-pkor1-B1	*ggggacaagtttgtacaaaaaagcaggct*TGGAGACTGTTGAAAAGCCG	tagN deletion in WH39
tagN-pkor1-B2	*agtgtcttgatttgtacgtca*TTGCATAAGGGCTATCCGTG	tagN deletion in WH39
tagN-pkor1-B3	*gacgtacaaatcaagacact*GGTGGGTTAACGGTAAAAGGC	tagN deletion in WH39
tagN-pkor1-B4	*ggggaccactttgtacaagaaagctgggt*AGGCTATTTTGGGTTTCACTCG	tagN deletion in WH39
c-tagN-F	ACCGAAAGCTTTGCTGGATCCATGAAAAAATTTCGAAAAGTAATTAAGAA	*tagN* complementation
c-tagN-R	TGGTGATGAGAACCTCTCGAGCCAATAAATTATAAAATTCAGACATAGCTTTC	*tagN* complementation

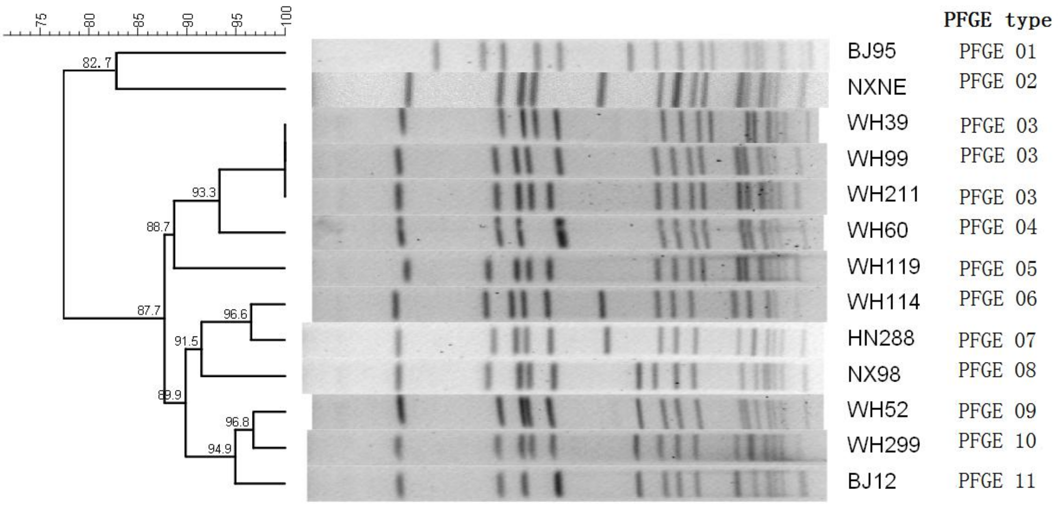

Figure S1 Cluster analysis of 13 clinical ST630 *S. aureus* strains by PFGE.

(a)

```
S. aureus WH39              ATGACTATGAAAATATTAATAACAGGTGGAGCTGGATTTATTGGTTCGCATATTGCTGAATATTTTGTAGAACATTACAACGATGTTTATATAATAGATAATTTAGTTTCAGGATATCGA
S. pseudintermedius ED99    ------ATGAAGATATTGATAACTGGTGGTGCTGGATTTATTGGTTCACATGTGGCAGAACTTTTTTTCAACCAACATGATGAAGTTTATGTTATGGATAATTTGAGTACGGGTTATCGA
S. epidermidis B1200343     ------TTGAAAATTTTAATAACTGGTGGAGCAGGTTTCATTGGATCACATATAGCTGAATTTTATGATTCTAATAGATATGAAGTTTTTATACTAGATAATTTAGAAACAGGAAAAAGG
                                  :****.**:**.*****:*****:**:**:** *****:**.***.* **:*** :**:* :   . .*: .   * **:****:*.*:.*.********.. ::*.**::*:.*.

S. aureus WH39              GAAAATGTTGATTTTATCAAGGATGAAAATTTTTTTAATATTGATGTAACTAATTATACAGAAGTTAATGAATTAATAGAAACATATCAATTTGATGTCATCATTCATTTGGCAGCTATG
S. pseudintermedius ED99    AAGAATATTCCTTTTATTGATGATGAACATTTCTTACATATCGATGTTAGAAATCATGAAGCAGTGGCGCATATCATACAAACGCATCAATACGATATCATTATCCATTTGGCATCAGTC
S. epidermidis B1200343     GAAAATGTTCACTTTATTGACGAAAAACACTTCTTCAATGAGAGTGTAACAAATCAATTATTTATTGAAAATCTTATTAAAAAATATCAATTTGATATAGTAATTCATTTAGCAGCAGTT
                            .*.***.** . ***** .* **:.**.* ** ** .**.: ..***:* :*** *:  *  :.* ..  *: * **: ***.. ******: ***.*..* ** *****.*** *:.*

S. aureus WH39              GTAAGTGTAGTGGAGACTGTTGAAAAGCCGGAACAGTCAAATAAAGTTAATATTGATGCAGTTTTAAATATATTACAAGCATGTCGTGAAAAAAATCCTGGTATAAAAAAGATTATATTT
S. pseudintermedius ED99    GTCAGCGTCGTAGAGACGATAGAAAATCCTGAAGTGTCCAATGAGGTCAATATTTCAGCACTTGTTCATTTACTTGAATTAAGTCGAAAACACTTACCTCATCTTAAAAAATTTATCTTC
S. epidermidis B1200343     GTAAATGTAGTAGATACAATAAACAATCCTATAACATCGCATAAAGTAAATATTAAAGGAACATTAGATATTTTAGAAGCAAATAAAAAATTTAACTCAAATCTTAAAAAATTTATTTTT
                            **.*. **.**.** ** .*:.*.** ** .:*  .** .**.*.** ****** .:* *  : *: **:*: *: **  *:.*..:.** : ::  *: .*.*:*****.:**** **

S. aureus WH39              GCATCTTCAGCTGCGGTTTATGGAAATGAAAAGTCATTACCTAAATCAACACATAGTCCAATACAACCTGAATCTCCATATGCAATACAAAAATATTGTGGAGAACAATATGTCAAACTG
S. pseudintermedius ED99    GCGTCTTCAGCGGCAGTTTATGGTAATAATCCGACTTTACCTAAAAGTATCACGAGTCATATTCAACCTGAATCTCCATATGCGATTCAAAAATATAGTGGTGAACAATATGTAAAGTTG
S. epidermidis B1200343     GCTTCTTCTGCAGCTATATATGGAAATACTGATTCTCTACCTAAACAAGTAGAATCATTTATTAATCCTGAATCTCCATATGCCATAGAAAAATATACTGGAGAACAATACACTAAACTA
                            ** *****:** ** .*:*****:***..: . :*: ********  :. . . : :  :**:.*:***************** **: ********: ***:******** .  **.*.

S. aureus WH39              TATAGTAGTCTCTATAACTTGCCCACAGTATCTTTAAGGTTTTTTAATGTTTATGGACCTAAACAAGATCCAAAATCACAATACTCAGGGGTTCTTTCAATCATTAAAAGTGCATTTGAG
S. pseudintermedius ED99    TATCATGACTTGTATCATTTACCGACGACGGCACTCCGATTTTTTAATGTTTACGGTCCTAAACAAGATCCTCAATCTCAATATTCAGGGGTACTCTCCATTATGAAACACTGTTTTGAT
S. epidermidis B1200343     TATTTTAATCTTTATAATTTACCAACTACATCATTAAGATTTTTTAATATTTTTGGTCCAAGACAAGACCCATCATCCCCATATTCTGGTGTGATTTCAATAATGAATGAAAAAATTTAAA
                            ***  *..  * ***.* **.** **  . . *: *..*.*********.***: **:**:*.****** **: .*** *.*** **:** ** .* **.** ** **: .   :***.*

S. aureus WH39              AAGGATGAAATATTCAACTTATATGGCGATGGGAAGCAAACAAGAGATTTTGTTTATGTAAAAGACGTTGTCCAAGCAGTGAAAATTGTATTGGAAAATGAAGGATGTAACGGCGGTGTA
S. pseudintermedius ED99    CAAGATGAACCATTTACTTTCTTTGGCGATGGTGAACAAACAAGAGATTTTGTTTATGTTAAAGATATCGTTCAAGCTATTCAAATTGTGATTCAACAACATATCTCGCATGGTGCTGTA
S. epidermidis B1200343     AATAACGAAAAATTTACATTTTTTGGTGACGGGAATCAAACTAGAGATTTTGTATATGTAAAAGATTTAGTTCAGGCTATAGACATAGTTATTCAAAATGATAATACAAAAGGACATATT
                            .* .* ***. *** *. ** *:*** ** ** .* *****:***********:*****:*****  * ** **.**:.*  *.**:** :*  **.*: *:.  :  .* **   *.*:

S. aureus WH39              TATAATGTTGGTACTGGAACTGCAACATCTTTATTAACAATAATTGATACTTTTTCAAAGCTAATAAACAAGCAAATTGAAATAAATTATCTCAATAGTAGAAAAGGTGATGTTAAATAT
S. pseudintermedius ED99    TACAATGTAGAGACGTCGCAACCCACATCTTTATTATCGATATATGAAATTTTTAAACATTTGTATCAAAAAGAAATTCCAACGCAATTTGCTGACCCAAGAGAAGGCGATGTGAAGTAT
S. epidermidis B1200343     TACAATTTAGGTACTGGTATTCCGTCTTCCTTAAAGGATATATTTGACAATTTTACTAATATATTTGACAAACAAATTGAATACAATCATTCTATTGAAAGAAAAGGAGATATTAAACAT
                            ** *** *:*. **     . : * :*:** ***:.. . ***::*** * ****:..:.*  *.::: *.**. ***** .*:  .*: :*   .:   :***.**** ***.* **. **

S. aureus WH39              TCATATGCCGATATTACAGAACTAAAAGCCTTTGGGTATACACCAATGTATAGCATTGGCAAAGGATTGAATGAATACTTAGAATAT---AGTAATCGATTTATAAATTAG
S. pseudintermedius ED99    TCGTATGCGGATATTGAACCATTAAAACAATTGGGGTTTTCAACGCAATATTCACTAGAAGAAGGACTTAATGAATATTTAAATTATGAAATGTATCATCCTTTATCATAA
S. epidermidis B1200343     TCATATGCGAATATTGATAGTTTATCTTCTCTAGGATATAAACCAGAATACACTATTGAAAAAGGACTACTAGAGTATG--------------TTCATTCACAGTAA---
                            **.***** .*****..:  : **:.: .   * **.*:*:.*.*. :.** :  .*:*...***** * .::**.**               :**.:  : :.:.:
```

(b)

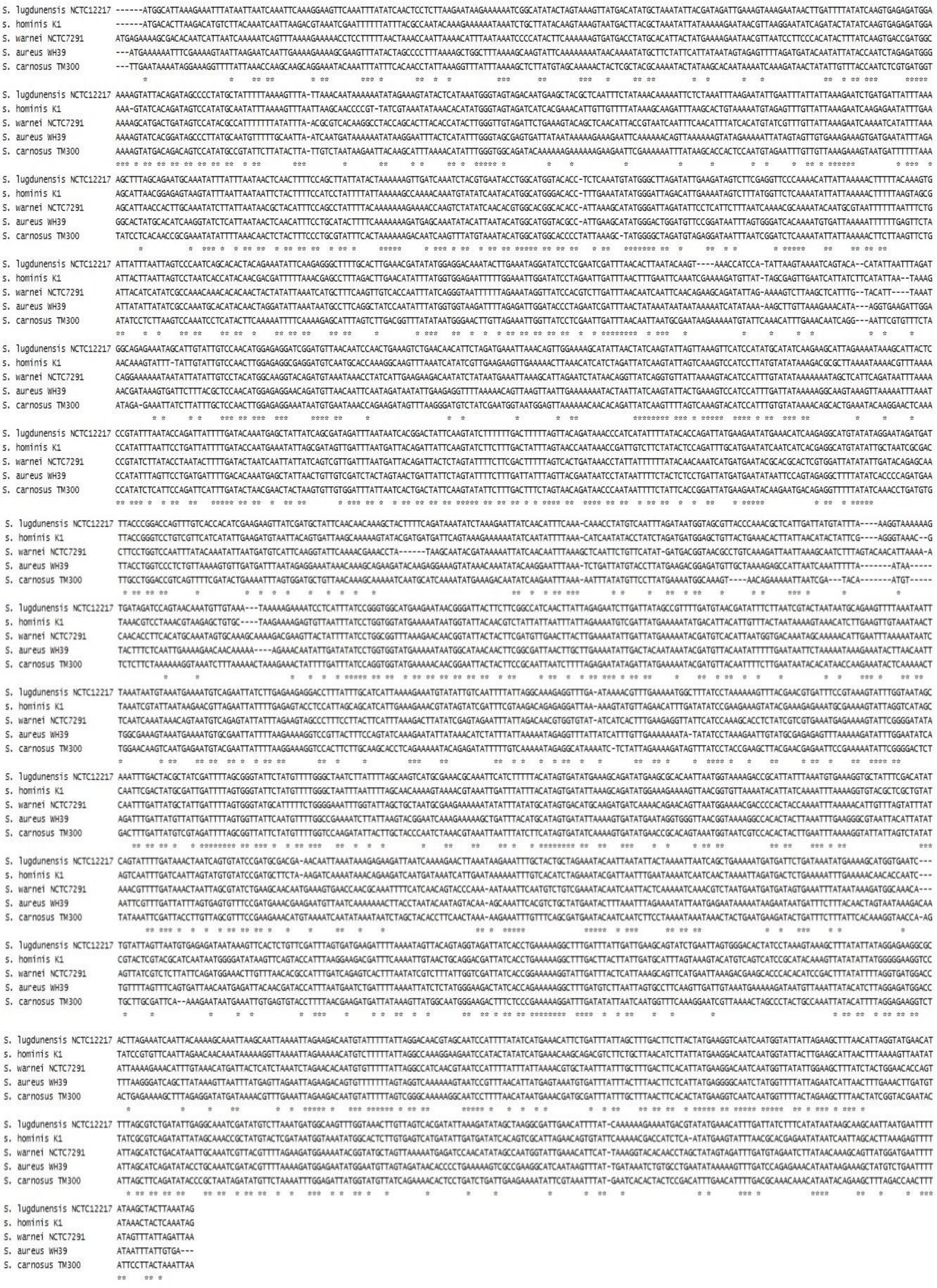
S. lugdunensis NCTC12217
S. hominis K1
S. warnei NCTC7291
S. aureus WH39
S. carnosus TM300

(c)

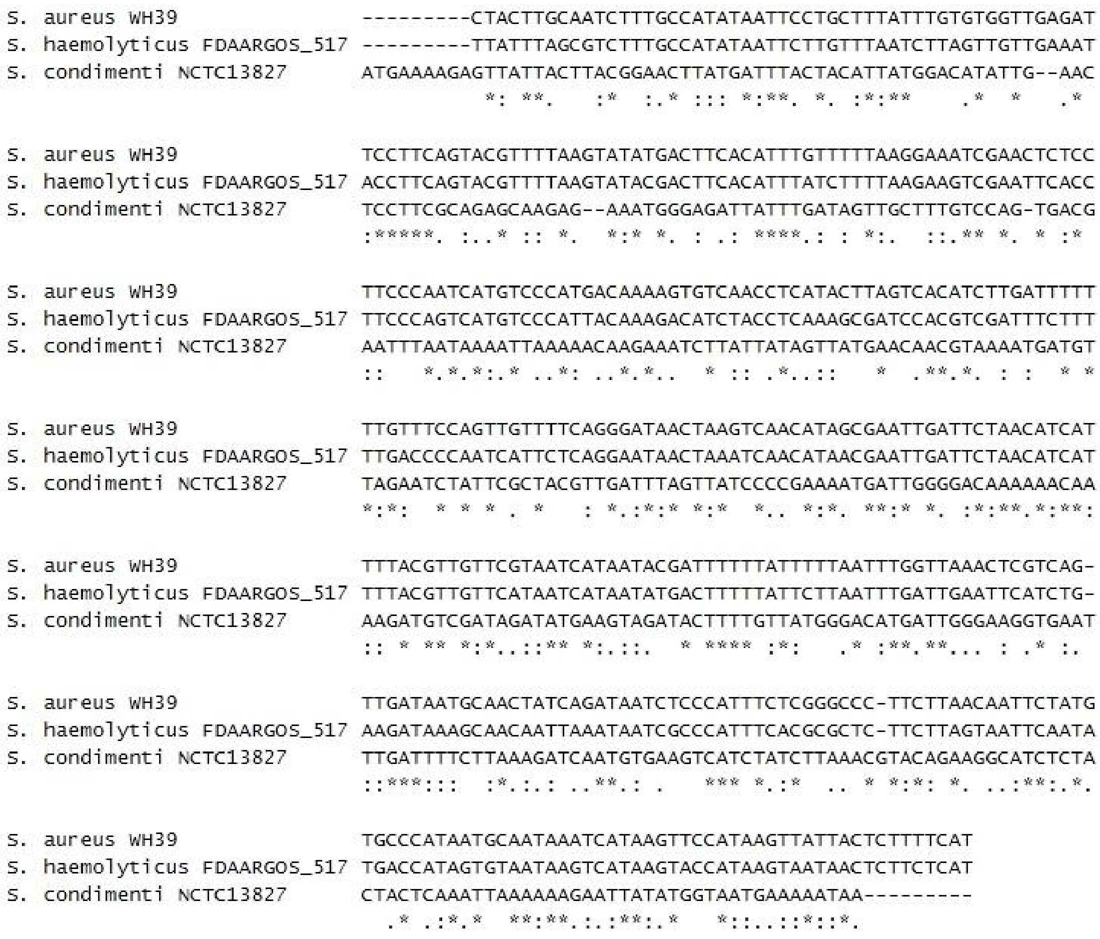
S. aureus WH39
S. haemolyticus FDAARGOS_517
S. condimenti NCTC13827

(d)

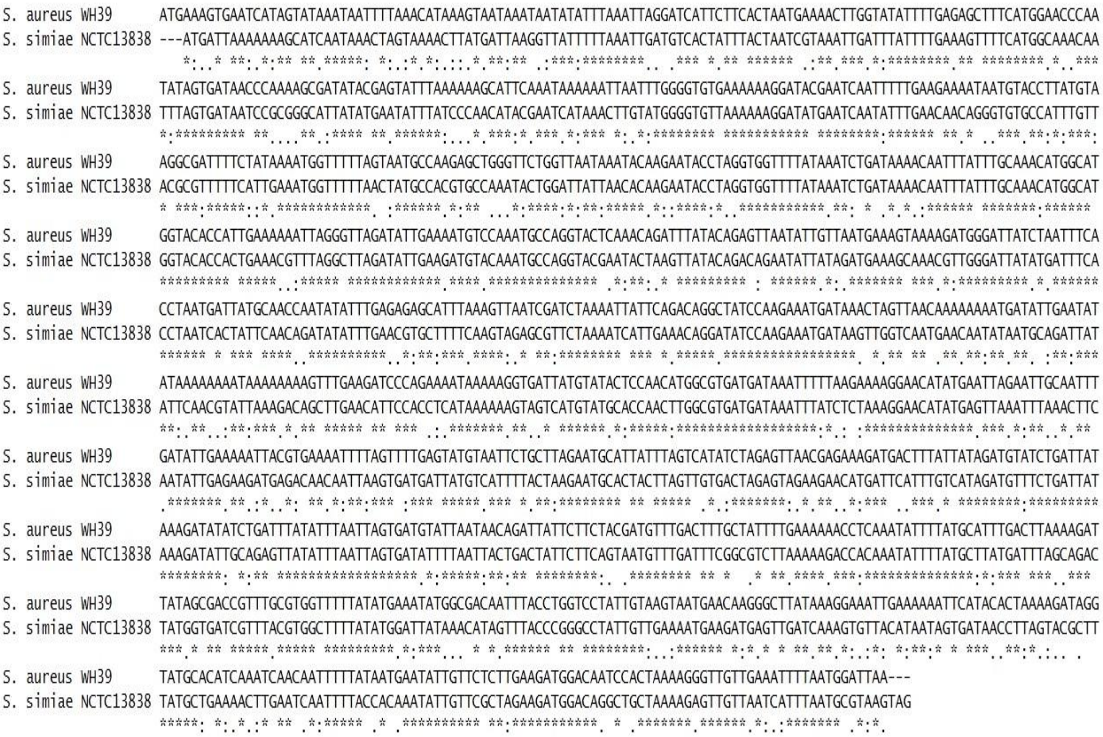
S. aureus WH39
S. simiae NCTC13838

Figure S2 Gene sequence alignment of (a) *tagV* with its close homologue identified in *S.pseudintermedius* ED99 (69.11% identity) and *S. epidermidis* B1200343 (69.11% identity); (b) *tagN* with its close homologue identified in *S. carnosus* TM300 (68.77% identity), *S. lugdunensis* NCTC12217 (65.25% identity), *S. hominis* K1 (65.01% identity), and *S. warnei* NCTC7291 (64.67% identity); (c) *tagD* with its close homologue identified in *S. haemolyticus* FDAARGOS_517(80.56% identity) and *S. condimenti* NCTC13827 (80.98% identity); and (d) *tagF* with its close homologue identified in *S. simiae* NCTC13838 (72.12% identity). The homology searches and alignments were performed using blastp and ClustalW. Sequence identity (*),strong similarity (:), weak similarity (.) and difference ( ) are indicated.

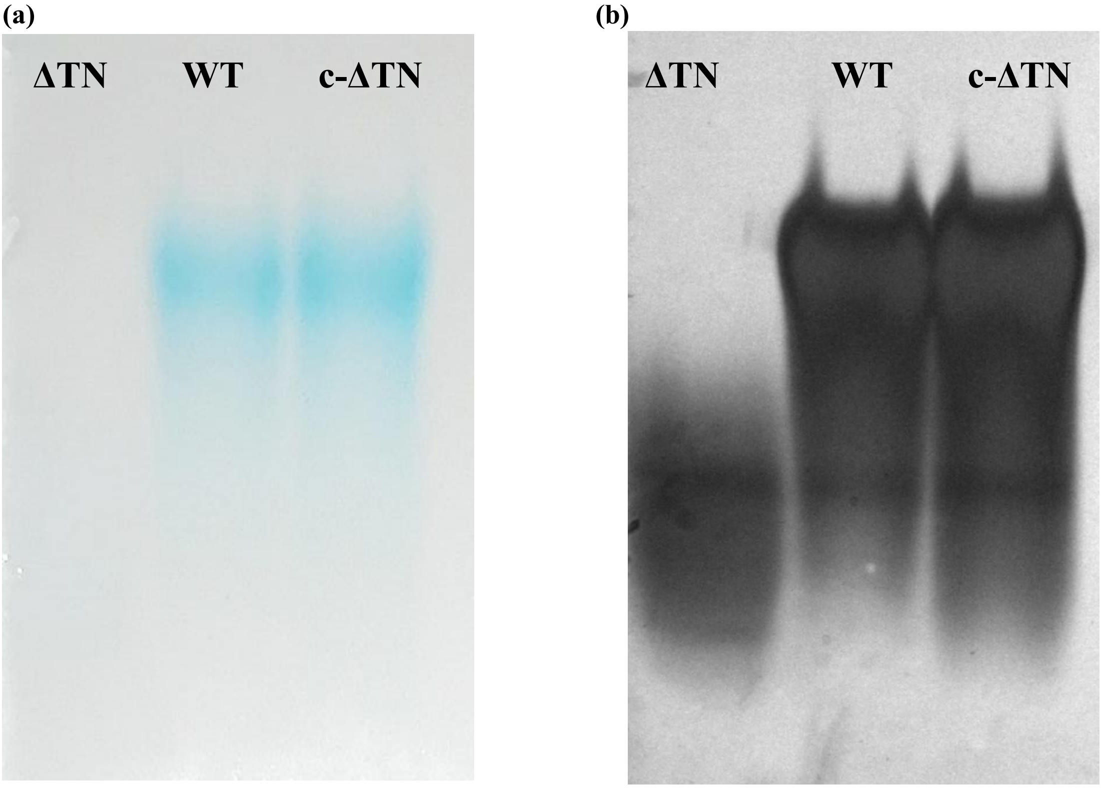

Figure S3 Native PAGE analysis of WTA preparations of *tagN* mutant (ΔTN) and its complementation (c-ΔTN) in WH39. Samples were resolved in polyacrylamide gels and visualized with alcian blue (a) /silver (b) staining.
